# Adaptation and validation of the Inventory of Family Protective Factors for the Portuguese culture

**DOI:** 10.1590/0104-1169.3315.2509

**Published:** 2014

**Authors:** Cláudia Cristina Vieira Carvalho de Oliveira Ferreira Augusto, Beatriz Rodrigues Araújo, Vítor Manuel Costa Pereira Rodrigues, Maria do Céu Aguiar Barbieri de Figueiredo

**Affiliations:** 1 Doctoral student, Instituto de Ciências Biomédicas Abel Salazar, Universidade do Porto, Porto, Portugal. Assistant, Escola Superior de Enfermagem, Universidade do Minho, Minho, Portugal; 2 PhD, Associate Professor, Instituto de Ciências da Saúde, Universidade Católica Portuguesa, Lisboa, Portugal; 3 PhD, Coordinator Professor, Escola Superior de Enfermagem de Vila Real, Universidade de Trás-Os-Montes e Alto Douro, Vila Real, Portugal; 4 PhD, Coordinator Professor, Escola Superior de Enfermagem do Porto, Porto, Portugal

**Keywords:** Family, Resilience, Psychological, Validity of Tests

## Abstract

**OBJECTIVES:**

to adapt and validate the Inventory of Family Protective Factors (IFPF) for the Portuguese culture. This instrument assesses protective factors that contribute to family resilience. Studies addressing resilience are embedded within the salutogenic paradigm, i.e. it addresses protective factors of individuals or groups without underestimating risk factors or vulnerability.

**METHOD:**

in order to assess the IFPF's linguistic and conceptual equivalence, the instrument was translated, retro-translated and the think-aloud protocol was used. We then verified the instrument's sensitiveness, reliability and validity of results to assess its psychometric characteristics. A factor analysis was performed of the principal components with varimax rotation of the scale's items and Cronbach's alpha coefficient was calculated for each dimension. A total of 85 families with disabled children, selected through simple random sampling, self-administered the instrument.

**RESULTS:**

the IFPF presents psychometric characteristics that are appropriate for the Portuguese population (Cronbach's alpha = .90).

**CONCLUSION:**

the IFPF was adapted and validated for the Portuguese culture and is an instrument to be used in studies intended to assess protective factors of family resilience.

## Introduction

One of the issues currently emerging within the scientific community, particularly among healthcare and education workers, is that certain families are not only able to respond positively to adversities and cope with them, but also to become stronger, optimistic and feel renewed and positively transformed by these situations. Resilience is the ability to overcome a potentially traumatic situation and regain strength, which implies positive adaptation to hardships and normal development, despite risk factors, and self-control after a traumatic event^(^ 1 ^)^.

The first studies exploring the concept of resilience focused on individuals' personal characteristics and coping strategies (adult or child) used to face adversities. One of the first studies addressing adaptive responses to adverse situations was developed in the 1970s with children at high risk. These children did not mirror the hardships they were subject to, but rather grew and became stronger than others in similar situations^(^ 1 ^)^. Research on resilience was extended to different age groups and different types of diversities, such as poverty and violence^(^ 2 ^)^, maltreatment^(^ 3 ^)^, and chronic diseases^(^ 4 ^)^. One group of researchers also investigated the relationship of this concept with cultural and ethnic characteristics of American and Hawaiian Indians^(^ 5 ^)^. These studies indicate a sense of resilience focused on personal attributes, such as autonomy and self-esteem.

Recently, some authors shifted the focus from personal resilience, previously based on individual resources, to a concept of family resilience, as a product of family relationships^(^ 6 ^)^. Family resilience is viewed as a family's ability to cultivate strengths that enable one to deal with changes in life. Underlying this concept are certain characteristics, dimensions and properties that ease adaptation of the family to change and crisis situations. This perspective acknowledges family strengths and dynamic relationships and considers that family stress and changes are not obstacles but an opportunity to grow^(^ 7 ^)^.

Families use coping strategies to deal with stressful situations in order to adapt. One has to consider the differences between resilience and coping. Resilience involves two processes: the first consists of resistance to stress and, therefore, ability to cope; and the second is more related to an ability to carry on with development and increase competences in an adverse situation^(^ 1 ^)^. Therefore, the focus of family resilience is on essential areas that enable family strengthening in the face of crisis situations, namely: (i) assigning a meaning to adversity; (ii) hope and optimism; (iii) spirituality, flexibility, cohesion, family communication, sharing leisure, routines and rituals; and (iv) support networks and family ability to maintain itself^(^ 7 ^-^ 8 ^)^.

Key processes of family resilience constitute family resilience based on the family adaptive resources, system of beliefs, patterns, and family organization, as well as communication processes(6). The identification and study of protective factors of families are important for nurses to perceive essential processes that help families to overcome transitions. Currently, the investigation on protective factors, based on the concept of resilience(7), has provided evidence that enables professionals and others to extract competences and potential of each individual or family as a whole and encourage an active process of restructuring and growth(6). This approach, which emphasizes family strengths rather than vulnerability and risk factors, is not apparent in most contexts of investigation and care practices. Hence, studies addressing family resilience are incipient, far from becoming strong studies with empirical evidence. For this reason, this is a broad field of investigation(7).

**Figure 1 -:**
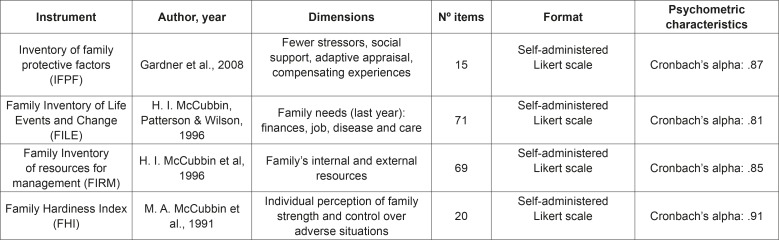
Characteristics of some assessment instruments with regard to their strengths and resources.

There are some instruments that permit assessing families from the perspective of their strengths, such as the Family Inventory of Resources for Management^(^ 8 ^)^, Family Hardiness Index^(^ 9 ^)^, Family Resource Scale^(^ 10 ^)^, and Inventory of Family Protective Factors^(^ 11 ^)^. Figure 1 presents the main characteristics of these instruments with regard to the dimensions assessed, number of items, format and psychometric characteristics.

Some of these instruments are not frequently used by professionals, given their complexity and especially because of the time required for application. We selected the Inventory of Family Protective Factors (IFPF)^(^ 11 ^)^ for this study because it enables professionals to rapidly assess families' protective factors that contribute to family resilience. The IFPF was developed and validated by five American researchers from the Lehigh University, New Mexico State University and University of Wisconsin - River Falls based on the Family Adaptation Model^(^ 12 ^)^. In this context, protective factors are assessed as opposed to risk factors, meaning that certain families have some attributes and resources that enable them to overcome and take advantage of demands inherent to transition processes, whether these are developmental or situational processes^(^ 6 ^)^.

**Figure 2 -:**
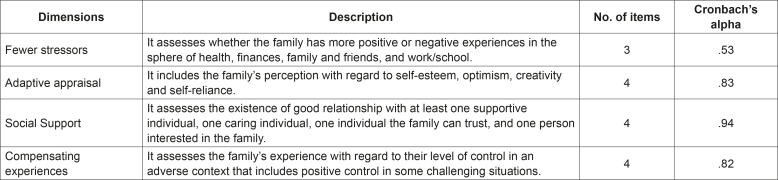
Dimensions of the Inventory of Family Protective Factors: description, number of items, and Cronbach's alpha values.

The Inventory of Family Protective Factors (IFPF) assesses four dimensions that influence family protection: fewer stressors, adaptive appraisal, social support, and compensating experiences, as described in Figure 2. The Cronbach's alpha for the instrument as a whole was .88. The coefficients obtained in the original version, as shown in Figure 2, in general suggest good internal consistency for the four dimensions of IFPF (equal to or higher than .70) with the exception of the "fewer stressors" dimension.

Items are scored on a 5-point Likert scale: (1) Almost always; (2) Generally; (3) Sometimes; (4) A little; (5) Not at all. The instrument's maximum score is 75 and the minimum score is 15. The procedures to construct the IFPF ensure an instrument with appropriate parameters of sensitiveness, reliability and validity. In order to assess the IFPF's psychometric characteristics of reliability and construct validity for the Portuguese population, we applied this instrument among families with disabled children. We consider that disabilities are a condition that imposes irreversible changes in the lives of children and families, which becomes a multidimensional experience for both the child and family^(^ 13 ^)^.

Given the previous discussion, this study's objective was to adapt and validate the IFPF to the Portuguese Culture, considering the availability of a multidimensional instrument that permits assessing protective factors that contribute to family resilience and which can be used by nurses and other professionals in the fields of health and education.

## Method

For the adaptation and validation process of the measurement instrument, we adopted a theoretical-methodological framework^(^ 14 ^)^ that comprises both a qualitative and quantitative analysis of items. 

With regard to the qualitative analysis, we performed the procedures required for linguistic and conceptual equivalence. After contacting the instrument's authors, we learned that this instrument had never been used for the Portuguese population and were authorized to initiate the scale's validation process. Linguistic equivalence was first achieved with translation performed by two bilingual individuals, one nurse and one psychologist. They were chosen because they mastered the language and were familiar with both the field of study and the selected sample. After translation, the two versions were compared and, as there were no significant differences, the Portuguese version was retro-translated by a third translator who was unaware of the original version. All the versions were compared (original, translation, retroversion) and no significant disagreements were found. This version was sent to the authors to assess equivalence of the English language of each item, who authorized its use.

Afterwards, we proceeded to the instrument's conceptual equivalence. Hence, the final version was submitted to a committee of five judges: three nurses with experience in family health, one family and general practitioner, and one psychologist with background in family therapy, to analyze the instrument and suggest small adjustments in terms of clarity and understanding of instructions. 

The process of qualitative analysis was concluded after using the think-aloud protocol with a set of five families similar to the study's sample. At this point, we applied the instrument and recorded all the subjects' verbalizations. As a result, we obtained a sense of the instrument's format and visual appearance, understanding of instructions, understanding of different items, receptiveness and adherence to the content. At the end of this linguistic and conceptual analysis, we obtained a draft version of the instrument in Portuguese, which we applied to the study's sample. Afterwards, we proceeded to the quantitative analysis of items.

In this second analysis, we assessed the instrument's psychometric characteristics through verification of precision and reliability and validity of results.

Cronbach's alpha coefficient of internal consistency was used to test the reliability of each dimension. For construct validity, we performed factor analysis of the principal components with varimax rotation for the scale's items to identify underlying factors. The KMO test and Bartlett's test of sphericity were used to assess sampling adequacy for factor analysis. For the factor analysis to be harmonious and reliable, we established that loading below 40% would not be accepted. 

The study was conducted in Braga, Portugal in six facilities that integrate the Early Childhood Intervention National System (SNIPI) and which, therefore, provide integral support centered on the child and family, including preventive and rehabilitation measures in the education, health, and social spheres. To establish the sample size, we used the validation criterion that recommends five participants for each scale item^(^ 15 ^)^, which resulted in at least 75 individuals. Therefore, 85 families with disabled children were selected using simple random sampling. Through SNIPI we accessed 85 families who self-administered the instrument in the respective facilities. Data were collected between September and December 2011 and analyzed using IBM SPSS Statistics for Windows, version 20.0.

Ethical aspects were complied with and written authorizations were provided by the facilities participating in the study. The families also provided written consent and had their confidentiality, privacy, and anonymity ensured. Finally, due to the nature of the study topic, we decided not to reveal the names of the facilities these families attended.

## Results

The participant families had children between three months and 18 years old, with an average of 8.5 years old. Male children (73%) with cerebral palsy (90%) predominated. In terms of education, 66% of the children attended mainstream schools, 23% attended facilities that exclusively focused on special education, and 11% remained at home and did not attend any type of school. These 11% of the sample basically correspond to children between 16 and 18 years old who had already concluded secondary education. These children remain at home, and, according to the parents, there is little provision of training and opportunities for development.

Nuclear families (77%) prevail in this study's sample, followed by extended families (13%). With regard to the families' origin, according to the Classification of Urban Areas (TIPAU), 47% were from a predominantly urban area, 33% from a moderately urban area, and 20% were from a predominantly rural area. Concerning the families' social status, according to the Graffar Classification System, 57% belonged to middle class, followed by 20% in lower middle class, and 17% in upper middle class. According to the Family Apgar, 81% of the families saw themselves as highly functional, 15% with moderate dysfunction, and 4% with marked dysfunction.

**Figure 3 -:**
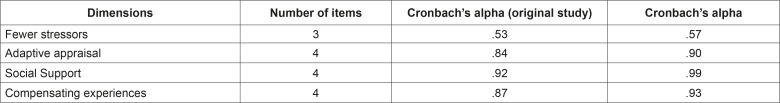
Dimensions, number of items, and IFPF Cronbach's alpha. Braga, Portugal, 2011

The Cronbach's alpha value found in this study for the instrument as a whole was .90, exceeding the original study, which found an alpha coefficient of .81^(^ 11 ^)^. Figure 3 presents the Cronbach's alpha values by dimension and number of items.

The Cronbach's alpha values suggest good internal consistency in the IFPF's four dimensions (equal to or higher than .60), except for the dimension "Fewer stressors", which presented a low coefficient (.57), but which we considered since it was higher than the one observed in the original study (.53).

**Figure 4 -:**
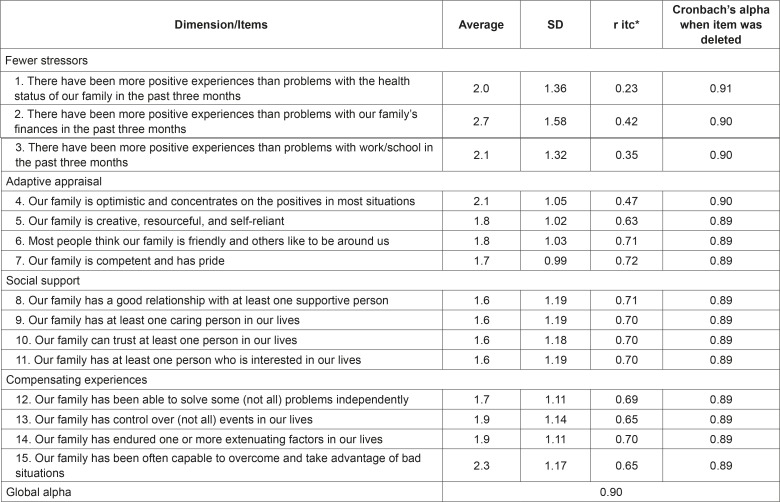
**Results of the Analysis of IFPF's Internal Consistency (n=85). Braga, Portugal, 2011** *r itc - coefficient of corrected item

Figure 4 presents the correlations of each item with the IFPF's Total Index of Intensity and Cronbach's alpha when the respective item was deleted.

The global Cronbach's alpha coefficient for deleted items ranged from 0.89 to 0.90, showing that the items jointly and equally contributed to the assessment of the construct.

Four factors were identified in the analysis of the IFPF's dimensionality performed by the authors of the original scale using exploratory factor analysis, which resulted from the application of the scale at three different points in time, namely: factor 1 - fewer stressors (items 1-4, item 3 was deleted); factor 2 - adaptive appraisal (items 5-8); factor 3 -social support (items 9-12), and factor 4 - compensating experiences (items 13-16). These four factors together explain 66.9% of the variance of the results in the four dimensions.

**Figure 5 -:**
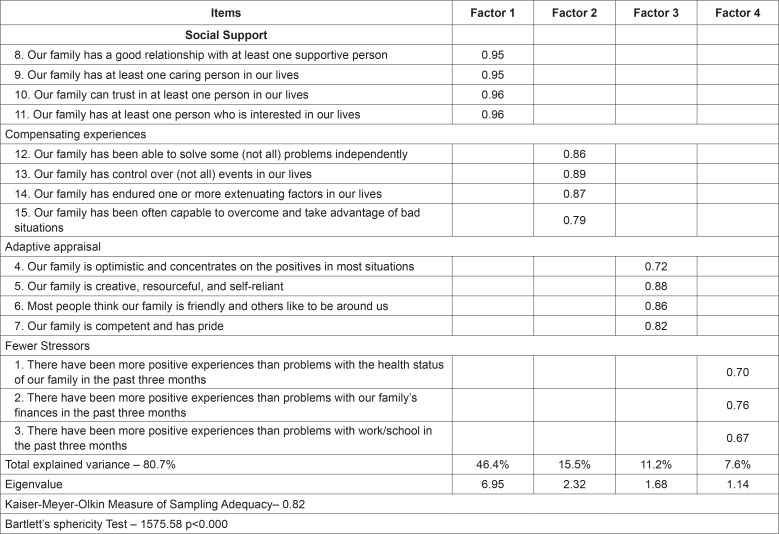
Results of the factor analysis of the principal components of IFPF. Braga, Portugal, 2011

With regard to the construct validity, the IFPF's items were submitted to factor analysis of principal components, as shown in Figure 5..

We initially performed the factor analysis without pre-establishing the number of factors, with varimax rotation and eigenvalue 1. The Kaiser-Meyer-Olkin (KMO) test and Bartlett's sphericity test (BST <0.05) permitted assessing the sample's adequacy for factor analysis (KMO = 0.82; BST = 1575.58, p <0.000). Hence, the factor analysis revealed four factors explaining 80.7% of the total variance. Even though there is a relationship among the items from a theoretical point of view, we note that none of the items significantly loaded in more than one factor. More specifically, after varimax rotation: factor I, which is related to social support and assesses the existence of a relationship with at least one supportive, caring, trustful person, and someone who is interested in the family, explains 46.4% of the total variance; factor II, which corresponds to compensating experiences and assesses the experience of the family in a context of adversity, explains 15.5% of the total variance; factor III, regarding adaptive appraisal that includes the perception of the family in regard to its self-esteem, optimism, creativity and self-reliance, explains 11.2% of the total variance; factor IV, related to fewer stressors, assesses whether the family perceives more positive or negative experiences in the health sphere, in terms of finances, family and friends, and work or school, and explains 7.6% of the total variance.

The results from the analysis of internal consistency indicate that the values of each of the four factors found in the factor analysis present good internal consistency indexes, with Cronbach's alpha values ranging from 0.57 to 0.93.

## Discussion

This study's results show that the IFPF presents appropriate psychometric characteristics to be used in the Portuguese population of families of children with disabilities.

The fewer stressors dimension presented a low coefficient of internal consistency that might be related to the fact that the items in this dimension, as opposed to the other dimensions, were restricted to an assessment of the last three months. This limitation was also observed by the authors of the original study, who chose to keep this limitation of time because these items refer to potentially transitory situations, as is the case of health, finances, friends, and work/school. We used the same criterion and opted to keep the items taking as reference the last three months. We believe this aspect should be taken into account in future studies.

Statistical tests to validate the construct of protective factors that contribute to family resilience through the four dimensions: fewer stressors; adaptive appraisal; social support; and compensating experiences, show logical relationships and the contribution of the 15 items to the global scale. According to the Family Adaptation Model^(^ 12 ^)^ underlying the development of this inventory, adaptive appraisal, social support, and compensating experiences represent the process of family protection and interact with the fewer stressors dimension to predict adaptation. When a child with disabilities is born, the family mobilizes resources to maintain balance, assesses the situation, and uses problem-solving strategies and family coping. In this situation, healthcare workers, based on the context and the family's characteristics, can identify and advise the family to mobilize the resources necessary to the management of the adverse situation to which it is subject^(^ 16 ^)^.

Family resilience is a dynamic process: a family may mobilize resources to cope with a situation or adverse event and, in another situation, may not be able to cope mobilize such resources, which corroborates the opinion of authors who consider that the assessment of family resilience cannot be generalized over time^(^ 7 ^)^. Hence, we suggest that protective factors be monitored at different points in time and in different circumstances. The times of assessment among families with disabled children could coincide with developmental milestones, which may be delayed or never reached by these children, potentially generating anxiety in the parents.

## Final considerations

The IFPF Portuguese version is an instrument that can be used by nurses in the context of primary healthcare to assess protective factors that contribute to family resilience. We suggest that family resilience is addressed at the beginning of the nursing program, when family health is taught, to enable more efficient nursing interventions in this domain.

One of the limitations of IFPF is related with low internal consistency of the "fewer stressors" dimension, a situation also observed by the authors of the original instrument and which may be explained by the fact that there is a temporal limitation (last three months). It is an aspect that should be taken into account when assessing these items in the future.

The IFPF version adapted to Portuguese showed to be a reliable instrument, valid and sensitive to assess protective factors of resilience among families with disabled children and, for this reason, we recommend its use. 

In short, we believe this study and the validated instrument contribute to the adherence of professionals to family assessment, which can be accomplished in a brief but comprehensive and multidimensional manner, with emphasis on the resources and strengths of families. 
